# Current Techniques for Investigating the Brain Extracellular Space

**DOI:** 10.3389/fnins.2020.570750

**Published:** 2020-10-14

**Authors:** Federico N. Soria, Cristina Miguelez, Olga Peñagarikano, Jan Tønnesen

**Affiliations:** ^1^Achucarro Basque Center for Neuroscience, Leioa, Spain; ^2^Department of Neuroscience, Faculty of Medicine and Nursing, University of the Basque Country (UPV/EHU), Leioa, Spain; ^3^Department of Pharmacology, Faculty of Medicine and Nursing, University of the Basque Country (UPV/EHU), Leioa, Spain; ^4^Autonomic and Movement Disorders Unit, Neurodegenerative Diseases, Biocruces Health Research Institute, Barakaldo, Spain

**Keywords:** single particle tracking, STED microscopy, brain parenchyma, glymphatic system, super-resolution, real-time iontophoresis, electron microscopy, brain extracellular space

## Abstract

The brain extracellular space (ECS) is a continuous reticular compartment that lies between the cells of the brain. It is vast in extent relative to its resident cells, yet, at the same time the nano- to micrometer dimensions of its channels and reservoirs are commonly finer than the smallest cellular structures. Our conventional view of this compartment as largely static and of secondary importance for brain function is rapidly changing, and its active dynamic roles in signaling and metabolite clearance have come to the fore. It is further emerging that ECS microarchitecture is highly heterogeneous and dynamic and that ECS geometry and diffusional properties directly modulate local diffusional transport, down to the nanoscale around individual synapses. The ECS can therefore be considered an extremely complex and diverse compartment, where numerous physiological events are unfolding in parallel on spatial and temporal scales that span orders of magnitude, from milliseconds to hours, and from nanometers to centimeters. To further understand the physiological roles of the ECS and identify new ones, researchers can choose from a wide array of experimental techniques, which differ greatly in their applicability to a given sample and the type of data they produce. Here, we aim to provide a basic introduction to the available experimental techniques that have been applied to address the brain ECS, highlighting their main characteristics. We include current gold-standard techniques, as well as emerging cutting-edge modalities based on recent super-resolution microscopy. It is clear that each technique comes with unique strengths and limitations and that no single experimental method can unravel the unknown physiological roles of the brain ECS on its own.

## Introduction

More than a century ago, Santiago Ramon y Cajal discovered that neurons are separate, individual entities rather than part of a continuum, and thereby laid the foundation for the *Neuron Doctrine* (Ramon and Cajal, 1888). While this doctrine prevails today, the last 20 years have seen a shift from the neuro-centric view of the nervous system toward a more integral scene that increasingly includes non-neuronal glial cells, in recognition of their unique and essential functional properties [reviewed in (Clarke and Barres, 2013; Araque et al., 2014)], and the fact that they constitute around half the neural cells of the brain (Azevedo et al., 2009). Even more recently, new functional roles of the brain extracellular space (ECS) and its constituent microenvironment have emerged or been hypothesized, with the ECS thereby claiming its place among the intricate ensemble of modulators of neural function. The ECS has recently been referred to as the *final frontier in neuroscience* (Nicholson and Hrabětová, 2017), eluding to its many unknown aspects and potential for new neurobiological discoveries.

The ECS must inevitably resemble a cast of its cellular constituents, a comparatively vast reticulum mirroring their extreme morphological complexity in the form of interwoven sheets, tubes, and bulbs that range over multiple orders of magnitude in size. While it is in one sense an enormous, brain-wide structure, it is in reality more difficult to visualize and study than its individual resident cells, because the substructures of the dense ECS meshwork are commonly only nanometers wide, far smaller than most cellular substructures. This fact is a major obstacle for researchers and a key factor when considering which technique to apply for addressing it.

It contains the diverse glycans and proteins of the extracellular matrix, which constitutes a hygroscopic plastic scaffold for patency and cell attachment. Yet, for the reason mentioned immediately above, the relationship between the structure of the ECS and that of the extracellular matrix is still to be explored. At the same time, all vacant ECS is filled by interstitial fluid (ISF), and as such, the ISF compartment directly corresponds to the ECS in terms of geometric structure. The ISF serves as transport medium for diffusing ions and cellular signaling molecules, and its constituent solutes mirror ongoing signaling and metabolic processes (Holter et al., 2017; Abbott et al., 2018).

Given that cells are separate entities, the ECS is involved in any and all signaling events taking place between cells of the brain, *sauf* that through cellular junctions. Synaptic neurotransmitters traverse the ECS at the synaptic cleft that is only around 25 nm wide (Lucić et al., 2005; Zuber et al., 2005), leaving limited space and time for the ECS compartment to putatively shape the post-synaptic response. However, in addition to such fast synaptic signaling, parallel events occur through *volume transmission* where signaling molecules travel up to several tens of microns through the ECS before reaching their target and exerting an effect (Agnati et al., 1986, 1995). The spread of these molecules will be modulated by ECS geometric shape and viscosity, spanning from the peri-synaptic level (Rusakov and Kullmann, 1998; Zheng et al., 2017), to the brain-wide glymphatic metabolite clearance system (Iliff et al., 2012, 2013a).

One of the most intriguing properties of the ECS is that it is structurally dynamic. It undergoes volumetric changes during hyperexcitatory discharge events (Colbourn et al., 2019) and has a circadian volume-change rhythm that facilitates metabolite clearance during sleep as part of the glymphatic system (Xie et al., 2013). Yet, even this state-of-the-art view represents a largely secondary, uniform role of the ECS with a conspicuous lack of regional specification. This, at least to some extent, reflects the technical limitations for experimentally addressing its structural complexity and dynamics, as we describe in more detail below. Consequently, we commonly describe the ECS in rudimentary terms. The ECS volume fraction (α) of total brain tissue volume is routinely reported as a ubiquitous value around 20% for a given brain area, or even the entire brain, though there must evidently be increasing variation as one zooms in on still smaller areas, with anything between 0 and 100% conceivable, and even inevitable, in submicron volumes. The same is true for the ECS tortuosity (λ), which is a quantification of hindrance to diffusion, and for the intercellular ECS gaps that separate neighboring cells and are currently accepted to be around 40 nm wide with limited variation (Nicholson and Hrabětová, 2017). These seeming generalizations and oversimplifications are rooted in the fact that most data on ECS properties arise from *volume-averaging* techniques, i.e., indirect approaches where the diffusion of extracellular probes is extrapolated and averaged from a relatively large volume of tissue. While indeed ECS parameters have been found to vary with cell type, developmental stage, and disease across studies (compared in Syková and Nicholson, 2008), the considerable differences in methodology and volume-averaging approaches between these studies are a genuine confounder when trying to provide a more comprehensive description of the ECS at any scale. Unfortunately, the requirement for volume-averaging has remained inescapable when investigating live tissue, due to the fact that the geometric structure of the ECS falls beyond what optical microscopy modalities can resolve, including high-resolution confocal and two-photon imaging approaches. We do have knowledge about the ECS meshwork dimensions from electron microscopy (EM), which readily provides nanoscale resolution images. However, EM is incompatible with live tissue imaging and diffusional measurements, and it has been associated with structural artifacts related to fixation procedures (Mishchenko et al., 2010; Korogod et al., 2015).

This highlights a key point: existing experimental techniques for investigating the ECS come with considerable trade-offs between live-cell compatibility, data dimensionality, and spatiotemporal imaging resolution, to mention just a few critical variables. Most notable among conventional techniques, there is a prominent disconnection between methodologies that offer sufficient spatial resolution to resolve ECS structure and the ones that are compatible with time-lapse imaging in live tissue, with limited options to bridge this gap. Only recently have developments in optical microscopy widened this bottleneck considerably and started to reveal the mesmerizing physiological structure and dynamics of the brain ECS in live tissue.

Our aim here is to present an up-to-date overview of the available techniques for experimentally addressing specifically the brain ECS, highlighting their individual strengths and weaknesses, as well as key insights they have brought about. We are not intending to provide a comprehensive review of the physiology and biophysical parameters of the ECS, as others have done so recently (Nicholson and Hrabětová, 2017; Hrabetova et al., 2018). Since the relationship between the distribution and composition of the extracellular matrix and the respective ECS structure and function is still not well understood, we do not here include methods focusing on extracellular matrix molecules. The review is rounded off by identifying open questions that can now be answered through application of the most recently developed techniques.

## Point-Source Diffusion Measurement Techniques

### Real-Time Iontophoresis Combined With Ion-Selective Electrode Detection

**FIGURE 1:**
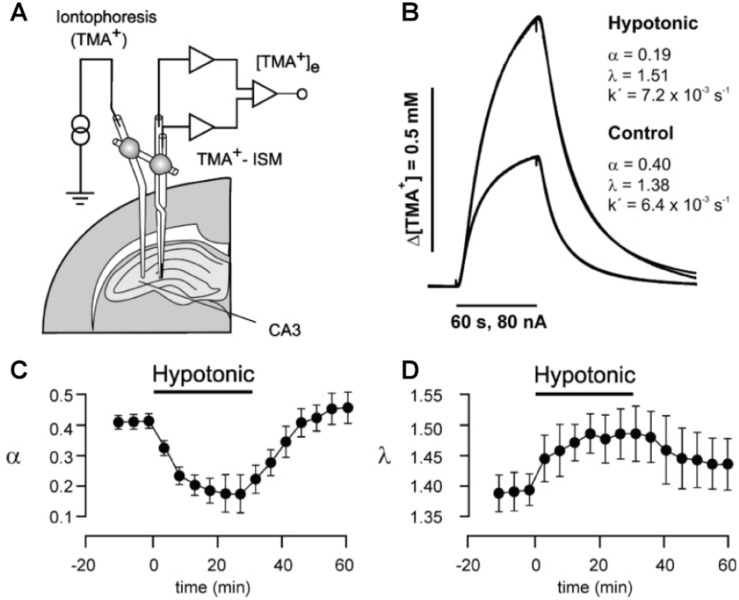
**(A)** Schematic drawing of setup for *in vivo* or slice real-time iontophoresis measurements of TMA^+^ diffusion in hippocampus. TMA^+^ is released iontophoretically from a thin capillary from where it spreads diffusionally and is measured by a TMA^+^ ion-selective microelectrode (ISM). **(B)** From the measured curves, the ECS volume fraction α and the tortuosity λ that quantifies hindrance to diffusion can be extracted, as here for respective hypotonic and control conditions in brain slices. **(C)** Change in α induced by transient perfusion of brain slice with hypotonic solution, measured by time-lapse RTI measurements. **(D)** Corresponding change in λ during hypotonic perfusion. As expected, hypotonic perfusion causes cells to swell, thereby reducing α and increasing λ. From Kilb et al. (2006), with permission.

This is the current gold-standard method for experimentally addressing ECS properties and is based on measuring diffusional spread of a molecule from a point-source, commonly in the form of physiologically inert tetra-methyl-ammonium (TMA^+^) introduced locally in the tissue *via* a glass capillary by *real-time iontophoresis (RTI)* (Nicholson and Phillips, 1981). Diffusional spread from the iontophoretic microelectrode source is measured by a corresponding TMA^+^ ion-selective microelectrode, which can be considered a point-detector, positioned at a chosen recording location, typically 100 μm away (Figure 1A). The RTI microelectrode capillary and the respective recording microelectrode can be positioned in the tissue independently or integrated in a single probe. The source/detector pair is calibrated for a given diffusion distance in aqueous agar prior to use, which represents a scenario of unhindered, homogeneous diffusion (Odackal et al., 2017). The technique is readily applied in live tissue, where it yields diffusion curve data (Figure 1B; Nicholson and Phillips, 1981). By comparing the measured diffusion curve in tissue to unhindered diffusion, it is possible to extract the ECS volume fraction, α, as well as tissue tortuosity, λ, as:

λ=√(D/freeD)tissue

where *D*_free_ is the diffusion coefficient in aqueous medium with osmolarity matched to brain ISF, and *D*_tissue_ is the diffusion coefficient experimentally measured in the tissue (Syková and Nicholson, 2008). The advantage of the RTI method is that it is live tissue compatible and can be performed in brain slices as well as *in vivo*. It allows repeated time-lapse measurements, which have been used to show that hypoosmolar conditions reduce α and increase λ (Figures 1C,D), and enhance epileptiform activity in the hippocampus of rodents (Kilb et al., 2006). By combining a single ion-selective recording electrode with two source iontophoresis microelectrodes, the method has further revealed anisotropic diffusion over 100 to 200 μm distances in the corpus callosum and hippocampus (Vorísek and Syková, 1997; Mazel et al., 1998). Through the RTI approach, α has been consistently reported around 20% across different brain areas, though ranging from around 10 up to 43% (Mazel et al., 1998; Syková and Nicholson, 2008), and with λ values around 1.3 to 1.8.

The most notable downside of the point-source/point-detector approach is that it does not visualize ECS structure and has very low spatial resolution relative to ECS structural geometries, defined as the 50 to 150 μm distance between the RTI capillary tip(s) and the recording electrode tip. This necessitates interpolation and averaging of tissue properties across the given probe distance, with physiological variation on smaller scales effectively inaccessible. While for a given probe distance, the exact position of the ion-selective electrode is not critical in homogeneous tissue, in areas with regional ECS variations arising from laminar tissue, such as in the hippocampus (McBain et al., 1990), or from 3D anisotropy, as in the cerebellum (Rice et al., 1993), detector placement becomes of significant importance. In these cases, where inhomogeneity exists on a distance similar to the spatial resolution of the method, the diffusion model needs to take such variation into account to correctly interpret ECS parameters, e.g., through a multilayer analysis (Saghyan et al., 2012).

Regarding temporal resolution, the electrical current pulses used for iontophoretic application of TMA^+^ are typically 1 min duration 100 nA square pulses (Odackal et al., 2017), and subsequent diffusional clearance of TMA^+^ from the area following the pulse takes a few minutes, thus enabling repeated sampling with an interval of 5 min. The technique was from the beginning introduced in an *in vivo* setting (Nicholson and Phillips, 1981), though a drawback is that *in vivo* RTI experiments are, to our knowledge, invariably performed in anesthetized animals. The putative effects of anesthesia have not been thoroughly explored, though no effects were reported in one study that did address it (Syková, 1997). Even so, it seems inevitable that anesthesia must have an effect on ECS properties given our current knowledge of brain functional changes in anesthetized animals and over the sleep–wake cycle (Xie et al., 2013; Stowell et al., 2019). The reported lack of anesthesia effects on measured diffusion could reflect limited sensitivity of the RTI method, which again relates to the required volume-averaging.

### Microfiber Optical Measurements

Microfiber imaging is another point-source approach, though it has not gained the popularity of the RTI-TMA method, and the existing publications have emerged from a single lab. In contrast to the RTI-TMA approach, it is optical rather than electrical in nature and measures fluorescence through the tip of an optical fiber inserted in the tissue area of interest. The fiber is used for both delivering excitation light and collecting emitted fluorescence from the illumination volume immediately around the tip, while a fluorophore is delivered iontophoretically or by pressure injection from a nearby capillary. Fluorescence intensity for a given excitation intensity and fluorophore concentration are measured in pure aqueous solution and inside the tissue, where the fluorophore solution distributes in the ECS. Given that the chosen fluorophore does not cross the cell membrane to enter cells, it will distribute exclusively in the ISF. A proportion of ISF is effectively displaced by cellular structures present within the tissue recording volume. Accordingly, the measured fluorescence intensity will scale with the volume fraction of the ISF and, thus, with ECS α. The method can be calibrated for a given experimental setting through cuvette experiments to provide quantitative measurements of α (Magzoub et al., 2009). For a given fluorophore concentration, tissue fluorescence scales roughly linearly with the volume displacement by cellular structures in the illuminated volume and thus with α (Zhang and Verkman, 2010). An extension of the approach is to combine it with transient fluorophore inactivation by photobleaching through high-intensity excitation illumination, followed by measurement of diffusional fluorescence recovery after photobleaching (FRAP). From the FRAP recording the re-equilibrium time constant can be readily obtained to reveal information about tissue diffusional properties, i.e., λ values across mouse brain areas (Zador et al., 2008). The technique resembles the RTI approach in many aspects, though instead of measuring diffusional equilibration electrically between two probes, it does so optically within an illumination volume. Similar to the RTI method, its main drawbacks include the lack of structural geometric ECS data and challenges in measuring from multiple areas, which becomes rather laborious, as it requires repositioning of the optical fiber and/or fluorophore-infusion capillary.

## Electron Microscopy

**FIGURE 2:**
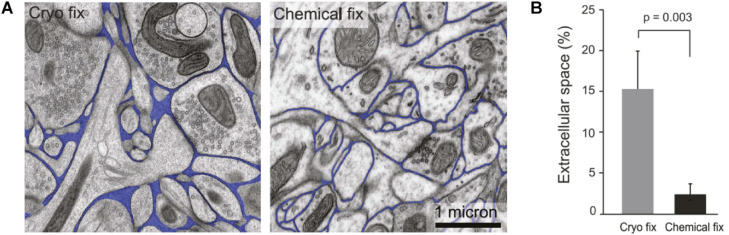
**(A)** Comparative images of cryo-fixed and chemically fixed brain tissue imaged by electron microscopy, with ECS indicated in blue. It is clear that cryofixation yields better preserved ECS that is more voluminous and heterogeneous in terms of intercellular distances. **(B)** Comparison of α assessed in brain tissue that is cryo-fixed and tissue that is chemically fixed. Cryofixation yields comparable results to RTI measurements, whereas chemical fixation causes cellular swelling and a severe underestimation of α. From Korogod et al. (2015), with permission.

At the opposite end of the spectrum to the point-source approaches, EM offers ECS images with nanoscale resolution and, until recently, has been the sole source of 2D and 3D images of the neuropil and ECS (Kasthuri et al., 2015; Korogod et al., 2015). Based on EM work, α has been estimated at an average 20%, in excellent agreement with the RTI-TMA method, and a predominant width of ECS channels at 40 nm (Nicholson and Hrabětová, 2017; Figure 2A). The major drawback of EM is the need for tissue fixation and inescapable incompatibility with live tissue work. The above-referenced data are based on high-pressure cryofixation-EM (cryofixation-EM) followed by freeze-substitution embedding, a technique pioneered more than 50 years ago to preserve water distribution and hence ECS ultrastructure (Vanharreveld et al., 1965). This methodology reduces structural fixation artifacts compared to chemical fixation (Figures 2A,B; Ohno et al., 2007; Korogod et al., 2015; Soria et al., 2020), though to some extent this confounder lingers, as it is still impossible to validate against a live tissue ground truth.

Classical chemical glutaraldehyde-based fixation dehydrates the sample and effectively shrinks the ECS, while cryofixation immobilizes molecules in their hydrated state without cross-linking, which has the additional advantage of preserving their antigenicity and therefore enabling concurrent immunolabeling protocols for correlative EM and fluorescence microscopy (van Rijnsoever et al., 2008). In cryofixation-EM, only the outermost tens of microns of a given tissue sample are reportedly artifact-free (Studer et al., 2008), and thicker tissue samples must accordingly be sliced prior to cryofixation. The initial steps of cryofixation-EM are therefore identical to acute brain slice work, such as that for electrophysiological recordings or fluorescence microscopy. In terms of invasiveness and tissue integrity, cryofixation-EM is therefore not superior to acute or cultured live slices when it comes to investigating cellular morphology in brain tissue. EM is still a superior method to fluorescence microscopy for connectomics studies and for quantifications based on counting, e.g. of synapses or synaptic vesicles, where the confounding effect of nanoscale distortions from fixation and slicing have a more negligible impact on results.

With respect to validation of the various existing and emerging techniques, it is gratifying that the range and distribution of ECS widths measured through cryofixation-EM in the mouse ventral midbrain are comparable to those recently obtained by super-resolution live-imaging methods applied in the same brain area (Soria et al., 2020), although a systematic per-region analysis is needed to further validate such findings.

## Magnetic Resonance Imaging

Magnetic resonance imaging (MRI) is a non-invasive approach extensively used for diagnostic purposes, as well as for clinical and experimental studies. The technique utilizes strong magnetic fields and radio waves to image protons in tissue, and thereby primarily maps out water and brain fatty tissue. Among the different implementations of this technique [for an overview of MRI subtypes, see (Runge et al., 2015)], diffusion-weighted MRI selectively maps out the random motion of protons and, thus, tissue water contents (Bammer, 2003). It can generate whole-brain 3D images at a spatial resolution up to 100 μm (Stucht et al., 2015), and while this is impressive considering the large field of view, it does not spatially resolve individual cells or individual ECS microstructures. MRI therefore also entails inevitable volume-averaging when analyzing ECS properties. Another notable confounder for studying the ECS is that both extra- and intracellular water will contribute to the MRI signal, and their respective contributions cannot be unambiguously separated (Norris, 2001).

To more specifically address the ECS compartment, some MRI studies rely on introduction of an extracellular contrast agent, e.g., based on hydrophilic gadolinium (Gd), which upon injection distributes by diffusion in the ISF and enhances the MRI signal by increasing the relaxation rate of water protons (Caravan et al., 1999). Comparison between MRI acquisitions before and after the contrast agent is introduced allows extraction of information more specifically related to ECS properties.

Intracerebroventricular infusion of a Gd-based contrast reagent in rats has been used to obtain distinct apparent diffusion coefficients (ADCs) for intra- and extracellular water and to further show that intracellular water is the main determinant of the overall MRI signal in both normal and ischemic rats (Silva et al., 2002). A similar approach, with introduction of the tracer either by intraparenchymal injection (Han et al., 2014) or by systemic injection followed by blood–brain barrier permeabilization *via* focused ultrasound (Mériaux et al., 2018), was used to extract ECS parameters such as *λ* and *D*_tissue_ in several regions of the rat brain. These approaches rely on the dynamic mapping of local concentrations of the tracer, followed by computation of ECS parameters using models that assume a constant flow rate of ISF in the whole brain and isotropic diffusion in the large volume scanned. While these assumptions may not hold true at the microscale, the method is very useful in that it maps coarse ECS parameters in 3D throughout the brain. In terms of imaging volume and image dimensionality, it outperforms the RTI-TMA method, though the RTI-TMA comes with superior specificity for the ECS compartment, as contrast-enhanced MRI signals inevitably incorporate confounding contributions from cellular and vascular compartments. A comparison study used MRI to map the movement of TMA and determine its ADC in the ECS (Kroenke et al., 2003). This was then compared to corresponding TMA ADC literature values obtained using the RTI-TMA method. The study revealed that the two techniques do not readily return comparable ADC values and that they are further sensitive to different biophysical parameters, which is not completely unexpected, based on the outlined differences between the techniques.

A free-water imaging (FWI) variant of MRI has been employed to enhance discrimination between extra- and intracellular water of brain tissue, by adding to the signal-processing algorithm a term that represents freely diffusing fluid. However, as this approach is still completely blind to the microscale geometry and absolute volume of the sampled ECS, the signal attributed to extracellular *free water* is not verifiable and is unlikely to represent true ECS values based on the limitations in spatial resolution and signal specificity of MRI in general. This FWI MRI technique, however, has been used in patients to identify an apparent increase in extracellular water associated with episodes of psychosis, which is hypothesized to reflect a neuro-inflammatory response and/or edema (Lyall et al., 2018; Tuozzo et al., 2018). A technique routinely employed in clinical settings, FWI MRI has further been used to demonstrate an increase in extracellular water content specifically in the *substantia nigra* of Parkinson’s disease patients (Ofori et al., 2015; Planetta et al., 2016), an area characterized by hallmark dopaminergic cell death in this disease.

**FIGURE 3:**
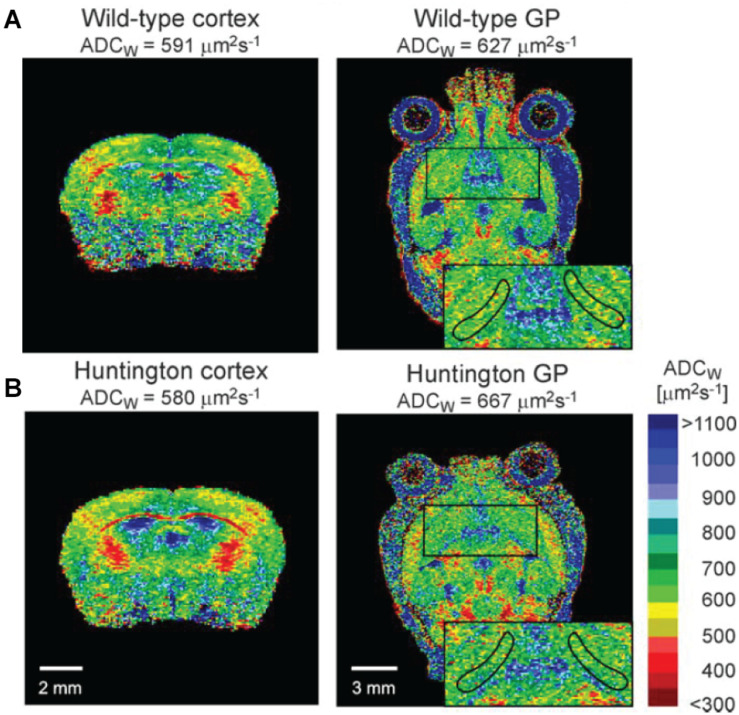
*In vivo* mouse brain MRI images depicted as the *apparent diffusion coefficient of water* (ADC_W_), as per the calibration bar. Extracellular water has a higher apparent diffusion coefficient than intracellular water, and the methods can be used to address differences in the extracellular space across conditions. **(A)** Depicts a coronal view and a sagittal view of the brain, including the cortex and the *globus pallidus* (GP; zoomed insert), respectively. **(B)** Depicts corresponding sections from the brain of a Huntington’s disease mouse model. It is apparent that water diffusion in the ventricles is much higher than in denser parenchyma, as expected. From Vorisek et al. (2017), with permission.

MRI has been applied to address molecular transport in cerebrospinal fluid, where the scale of the brain-wide 3D images is particularly suitable for addressing such transport as part of the glymphatic system. However, as we also describe, resolution and specificity instead become bottlenecks. In humans, intrathecal infusion of an MRI tracer revealed a brain-wide drainage of CSF to the cervical nodes, thereby corroborating the existence of a functional glymphatic system also in humans (Eide et al., 2018). The technique was previously employed to identify a reduction of rat brain ECS volume after cortical spreading depression (Hasegawa et al., 1995) and an increase in cortical ECS volume of aged transgenic APP23 Alzheimer’s disease model mice that was associated with the presence of cortical plaques and memory impairments (Syková et al., 2005). In the latter study, the authors presented parallel RTI data from the same animals that corroborated the increase in cortical ECS volume compared with controls. MRI has further been used to show an increase in α of the *globus pallidus* in experimental Huntington’s disease, consistent with observed cell death in this nucleus (Vorisek et al., 2017; Figure 3). The cell death and increased ECS volume were interestingly not associated with changes in diffusional properties of the tissue, which the authors attributed to concurrent astrogliosis and presence of reactive astrocytes, which could alter the tissue properties in a more complex way. More recently, MRI was applied to show that the presence of β-amyloid in the APP/PS1 Alzheimer’s disease mouse model was associated with reduced perivascular ISF flow and glymphatic system function. Notably, this effect could be effectively reversed through controlled heating of the brain tissue by transcranial illumination with red light over a period of 2 months, and this was further associated with rescue of impaired spatial learning (Yue et al., 2019).

While anesthesia is not a prerequisite for performing MRI, most MRI-based ECS studies to date rely on recordings from anesthetized animals, and this could conceivably influence tissue properties and confound data aiming to explore the normophysiological state. One MRI study in mice found that general anesthesia by ketamine injection or isoflurane inhalation impairs glymphatic system clearance and reduces ECS volume in a dose-dependent manner (Gakuba et al., 2018). This is partially supported by a more recent study in rats, where isoflurane inhalation anesthesia induced a decrease in ECS volume relative to intravenous anesthetics dexmedetomidine and sodium pentobarbital (Zhao et al., 2020). This latter work additionally found that norepinephrine had a negative effect on ECS volume and interstitial fluid drainage, corroborating original studies on the effect of noradrenergic signaling on ECS volume (Xie et al., 2013; Sherpa et al., 2016).

A related and complementary technique performed using the same equipment is *diffusion-weighted magnetic resonance spectroscopy* (DW-MRS), which relies on frequency analysis of the recorded magnetic resonance radio waves. In the detection band, a given frequency peak can be attributed to a given MR-observable molecule, e.g., water, glutamate, or creatine (Tognarelli et al., 2015). Thus, while MRI provides structural images predominantly based on water content of the tissue, DW-MRS provides maps of select biochemical components within a given voxel. The strength of DW-MRS is that it allows detection and discrimination of relatively specific cellular or extracellular species, such as from N-acetylaspartate that is nearly exclusively intracellular (Palombo et al., 2018). However, like MRI, it is ultimately a volume-averaging technique as it lacks the spatial resolution to resolve actual cellular and extracellular structures and is mostly used for qualitative analysis of tissue composition.

To recapitulate, MRI is a highly applicable tool for assessing differences in ECS volume and properties of glymphatic system clearance on a regional or global scale in live animals, where it can be repeatedly applied over periods of time spanning hours to years. The main drawback is the low spatial resolution and the problem in the separation of intracellular and extracellular signals, both of which prevent quantitative analyses of ECS volume fractions at any scale and prevent direct imaging of diffusion or flow of ISF or CSF. It is also not widely available to researchers due to the cost and complexity of the involved equipment.

## Widefield Fluorescence Microscopy

### Intrinsic Optical Signal Imaging

Intrinsic optical signal (IOS) imaging is in essence label-free light microscopy that detects changes in the tissue reflectance or transmission of light. As cellular membranes and the interstitial fluid have different refractive indices, light will be reflected and transmitted differently depending on whether photons are traversing cellular membranes, the intracellular or extracellular solute, respectively. The fractional volumes of cellular membranes and the interstitial fluid change with cellular activity, e.g., during cellular swelling, and will be reported as a change in the IOS (Frostig et al., 1995). However, the physics behind the observed signal are still not well understood, and the source of the IOS remains somewhat obscure, so the technique is limited at the level of its specificity to address the ECS (Tao et al., 2002).

The underlying microscopy technique is widefield imaging that can be readily performed at video speed and at a spatial resolution of a few microns. Label-free imaging is inherently associated with low contrast and individual neurons cannot be discerned in the IOS images, though the strength of the technique is exactly the simplicity, independence of labels, low phototoxicity, and the use of relatively simple widefield microscopes.

Classical IOS imaging detects reflected light and is thereby confined to address the surface of the brain, where it has been used to map out the functional architecture of the cortex, for example by enabling association of activity in specific barrel cortex areas to stimulation of individual whiskers in the rat (Grinvald et al., 1986). While the reliance on reflected light restricts experiments to the first few tens of micrometers of tissue surface, a variant of IOS imaging instead detects changes in transmitted light sent through the tissue of interest. This has been applied to study neuronal activity in unlabeled acute hippocampal slices non-invasively, showing that changing the ionic Cl^–^ gradient across the cell membrane *via* the loop-diuretic furosemide alters the detected signal (MacVicar and Hochman, 1991). As furosemide inhibits NKCC1 and KCC2 transporters predominantly found on astrocytes, this observation directly implicates astrocytes and their volume changes in the IOS and, thus, ECS volume changes (Andrew et al., 1999). In support of a primary role of glia in the IOS recordings, similar IOS changes in response to loop-diuretics furosemide and bumetanide have been observed in optic nerve preparations, which contain solely glia cells and axons, highlighting that the response is not depending on the presence of neuronal cell bodies (MacVicar et al., 2002). It has accordingly been argued that IOS primarily detects glia cell volume changes and, conversely, also ECS volume changes, which is supported by a positive correlation between ECS volume changes measured through extracellular TMA^+^ concentrations and the IOS signal, at least in the initial phase of the response (Holthoff and Witte, 1996). However, given that IOS changes are observed to be also dependent on neuronal activity and that loop-diuretics do not act exclusively on astrocytes, it is inevitable that neurons contribute to the signal too. Indeed, others have pointed out that the IOS changes are likely to involve neuronal components, including changes in mitochondrial membranes and dendritic beading in hyperexcitatory conditions (Aitken et al., 1999; Syková et al., 2003).

The simplicity of the technique, its *ex* and *in vivo* compatibility, and the imaging speed make IOS an attractive method for addressing cellular and ECS volume changes, though the lack of specificity and knowledge about the signal origin prevents quantitative ECS volume change analyses, thereby limiting the usefulness for studying specifically the ECS. The technique further has low spatial resolution, particularly in the *z*-axis when measuring transmitted light, and can only report tissue changes on a population level. It cannot visualize the actual ECS structure or discriminate between neurons and glia cells. Additionally, when applied in acute brain slices, the transmitted light changes report not only changes in the healthy middle layer of the slice, but also incorporate changes of the damaged top and bottom surfaces where the slice has been cut, which will add noise to the signal emerging from the healthy tissue layers.

### Integrative Optical Imaging

Integrative optical imaging (IOI) is based on widefield fluorescence microscopy and shares many aspects with IOS imaging described above. Though contrary to IOS imaging, the detected signal is not intrinsic, but instead derives from a designated fluorophore introduced by the experimenter. It is again live cell compatible, allows very fast CCD camera-enabled time-lapse acquisitions, and offers multicolor imaging, though at limited optical resolution and confined to the outermost tens to hundreds of micrometers of tissue. While the technique has low spatial resolution and is unable to resolve the geometric structure of the ECS in live tissue, it holds value for analyzing volume-averaged diffusional properties of larger volumes of tissue, e.g., across the field of view in a widefield microscope. It is based on acute introduction of an inert fluorophore into the tissue by pressure injection from a glass microcapillary tip and evaluation of the diffusional distribution of the fluorophore in the surrounding tissue. This allows diffusional ECS properties to be extracted, based on the static and dynamic fluorophore distribution around the capillary tip point-source. Acquisitions are easily performed using standard widefield microscopy cameras, and experimenters can readily extract data on the effective diffusion coefficient and λ at the resolution of the given microscope.

In the early days of brain ECS exploration, IOI was applied to show that λ increases with the molecular size of the probe (Nicholson and Tao, 1993). This important finding was achieved in rat cortical tissue using fluorescent dextrans of various molecular weights and later confirmed using quantum dots (Thorne and Nicholson, 2006), whose 35 nm diameter and ability to readily diffuse into the parenchyma reported that most ECS channels have at least 40 nm width. A recent development has improved the technique by increasing the temporal resolution from tens of seconds to around 1 s, to capture much faster ECS and tissue dynamics (Hrabe and Hrabetova, 2019). While in IOI the tissue diffusion coefficient (*D*) is assumed constant over time, in this improved *time-resolved IOI* version, *D* varies over time, allowing for detection of fast changes in diffusivity. It has been applied to show that diffusion in cortical ECS was greatly reduced during the depolarizing wave spread, consistent with a reduction in ECS volume (Hrabe and Hrabetova, 2019).

**FIGURE 4:**
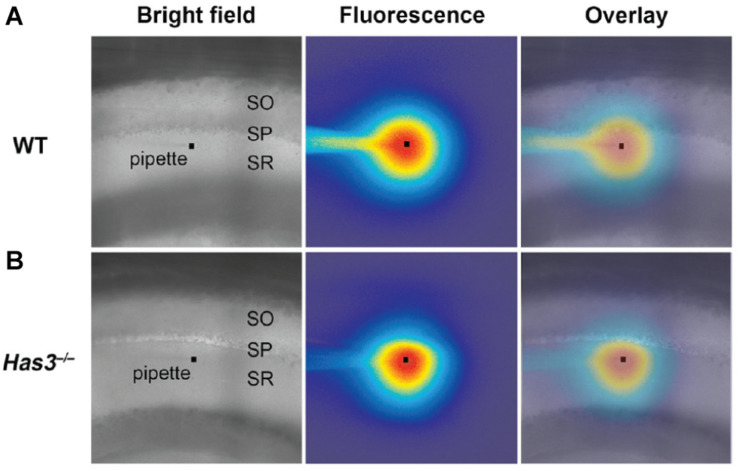
**(A)** Brightfield microscopy image of the CA1 area in acute brain slice from wild-type (WT) mouse. The *stratum oriens*, *stratum pyramidale*, and *stratum radiatum* layers are indicated as SO, SP, and SR, respectively. The faint outline of a micropipette is visible, with a square marking the tip. The middle panel is the same frame, though in epifluorescent mode 80 s after a brief injection of a fluorophore from the pipette tip. The diffusional spread of the fluorophore will depend on tissue diffusional properties, which can be read out by analyzing potential assymmetries in the fluorophore spreading profile. The right-most frame depicts an overlay of the frame imaged in brightfield and epiflorescence mode, repectively, enabling visual assessment of diffusion with respect to the indicated tissue layers. **(B)** Corresponding image from hyaluronan-deficient hyaluronan synthase-3 (Has3) knock-out mouse, where the fluorophore distribution around the pipete tip appears less symmetric, indicating assymmetric diffusion and changes ECS properties relative to the WT tissue. From Arranz et al. (2014), with permission. Copyright 2014 Society for Neuroscience.

Related to IOI, other widefield approaches have made use of fluorophore spreading after pressure injection to demonstrate heterogeneous diffusion across the molecular layer in turtle brain cerebellum (Xiao et al., 2008). Such heterogeneity was also reported across the layers of the hippocampal CA1 area in mice (Arranz et al., 2014), where it was increased after depleting the extracellular matrix protein hyaluronan through genetic knock-out (Figure 4). Much earlier, widefield phase contrast microscopy was applied to evaluate the effects altering interstitial fluid osmolarity on ECS volume in hippocampal slices, though here the readout was general histological appearance, not specifically that of the ECS (Newman et al., 1995).

The low cost, simplicity, and applicability of IOI make the approach attractive and readily available to researchers. The technique’s strength lies in the relatively high specificity of the fluorescence signal to the ECS compartment, as the fluorophore does not cross cell membranes to enter cells. This particular strength sets it apart from IOS imaging that suffers from low signal specificity. As a widefield microscopy modality, it comes with limited spatial resolution that does not allow ECS structure to be addressed, and it relies on extensive volume-averaging within the field of view, not least in the *z*-axis where optical resolution is lowest, commonly several tens of microns.

## Scanning Fluorescence Microscopy

### Two-Photon and Confocal Microscopy

Two-photon and confocal microscopy are highly similar optical point-scanning fluorescence microscopy approaches and, therefore, quite different in nature to the widefield modalities described above. Both come with inherently high optical *z*-axis resolution and superior optical sectioning that allows high-resolution imaging inside the tissue. Two-photon and confocal imaging modalities have been used to investigate fluorophore distribution and spread in the ECS, though not often in experiments actually intended to extract ECS physiological parameters. One such implementation was the use of two-photon microscopy to describe the concentration gradient of a membrane-impermeable fluorophore after pressure ejection from a fluorophore-filled microcapillary tip inside a brain slice, though the analysis was not intended to investigate the ECS compartment *per se* (Rusakov et al., 2005). Additional studies have used two-photon microscopy and point-source fluorophore introductions to analyze extracellular diffusivity of molecules, though without attempting to extract ECS geometrical data (Stroh et al., 2003; Savtchenko and Rusakov, 2005).

In (2008), Kitamura et al. performed local injection of fluorophores *in vivo* into the cerebral cortex ECS, as a way to visualize and patch the residing cell somata through two-photon shadow patching. This strategy utilized extracellular application of fluorophores to produce a negative shadow image of unlabeled cells through two-photon microscopy. While this approach directly renders the ECS fluorescent and visible, the diffraction-limited optical resolution of a two-photon microscope blurs most structural details out. This highlights the main drawback of two-photon microscopy for imaging the ECS; the best achievable resolution is around 400 nm laterally (*x*,*y*) and 1 μm axially (*z*), which allows larger dendrites and somata to be resolved faithfully, but not denser neuropil and its ECS geometry (Mishchenko et al., 2010). The optical resolution of confocal microcopy is around twice as good at 200 nm in the lateral plane and 500 nm axially. This resolution advantage over the two-photon modality is mainly due to the shorter wavelength excitation light and is only achievable if the setup is configured perfectly. Even then, this resolution is not sufficient to resolve the entire ECS geometric structure, though indeed many individual ECS channels already become visible and a lot of information is extractable. The shorter wavelengths of confocal microscopy make it less applicable for imaging deeper inside the tissue, and beyond a few tens of micrometers tissue depth, two-photon microscopy is a better choice and can be used to image down to roughly 1 mm depth, though spatial resolution will decrease at deeper layers.

**FIGURE 5:**
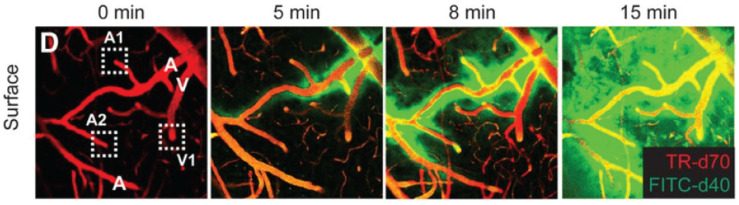
*In vivo* two-photon time-lape microscopy of blood vessels (in red), and a fluorophore spreading first in the perivascular space along arterial vessels (denoted A) and then into the parenchyma and along venules (denoted V), to effectively reveal the glymphatic system in function. From Iliff et al., 2013b, with permission.

Over recent years, two-photon microscopy has been increasingly used to study the glymphatic system, through its ability to visualize *in vivo* the distribution volume, wash-in rate, and wash-out rate, respectively, of fluorophores introduced into the interstitial fluid of the ECS through the ventricular system (Figure 5; Iliff et al., 2012, 2013b; Xie et al., 2013). This approach has been essential for our understanding of the glymphatic system as a metabolite clearance pathway, including the circadian volumetric change it undergoes to facilitate clearance during sleep, and the involved regulation through norepinephrine (Xie et al., 2013). In these studies, the resolution of the two-photon microscopy approach is sufficient to discriminate between perivascular spaces and denser parenchyma, though beyond this it offers no geometric information about ECS structure, as it cannot optically resolve this. A precursor work to these studies on the glymphatic system was a paper describing two-photon imaging of fluorescent nanospheres injected locally into cortical tissue, with concurrent imaging of cortical blood vessels in a second color (Foley et al., 2012). This provided the first images of the predominantly perivascular transport of solutes in brain tissue, though the term and concept of the glymphatic system had not yet been proposed. The use of nanospheres was more recently used to perform tracking of spheres along cortical arteries *in vivo*, to show that arterial pulsations drive perivascular cerebrospinal fluid bulk flow (Mestre et al., 2018).

It has further been shown that cortical spreading depression effectively impairs perivascular flow in a reversible way (Schain et al., 2017), consistent with cellular swelling that reduces the ECS volume (Charles and Brennan, 2009), as also reported using both the IOI and MRI methods above. In a mouse model of multiple cerebral microinfarcts by carotid cholesterol crystal injection, a global decrease in fluorophore spreading in the ECS was observed, indicating that focal disruptions of the glymphatic clearance pathway may have a global impact on putative bulk flow of ISF in a negatively age-dependent manner (Wang et al., 2017).

### Time-Resolved Fluorescence Anisotropy Imaging

Time-resolved fluorescence anisotropy imaging (TR-FAIM) allows optical assessment of viscosity by measuring the emitted fluorescence light polarization relative to the polarization of the excitation light. Polarized excitation light will preferentially excite fluorophores with a corresponding molecular orientation, and these emit fluorescence with a corresponding certain polarization. However, during the few nanoseconds between excitation and emission, the fluorophore will spontaneously undergo molecular rotation so that the emission polarization diverges from the excitation polarization; i.e., it is anisotropic. The level of anisotropy can be relatively easily quantified using two or more detectors with polarization-sensitive optical filters in front. The observed anisotropy depends on the viscosity of the medium in which the fluorophore resides, by impacting the degree of molecular rotation in a given nanosecond time window. Accordingly, differences in viscosity will give rise to differences in anisotropy, and in parallel to the optical fluorescence image produced by the microscope, a corresponding anisotropy map is produced (Le Grimellec et al., 1982). Fluorescence anisotropy imaging was originally utilized to measure cytosolic viscosity inside living cells (Dix and Verkman, 1990), though it has been adopted to measure viscosity in the extracellular space and even in the synaptic cleft by utilizing two-photon microscopy as the underlying microscopy modality (Zheng et al., 2017).

TR-FAIM has been applied to show that diffusion in the ECS is around 30% slower than in artificial cerebrospinal fluid, while in the synaptic cleft it is 46% slower. As two-photon microscopy is lacking optical resolution to actually resolve the synaptic cleft, this diffusional retardation measurement is incorporating considerable measurement noise and is conceivably substantially larger as well as heterogeneous within the cleft. The technique has been further used to show that viscosity of the interstitial fluid varies only slightly across the respective hippocampal CA1 *strati oriens*, *pyramidale*, and *radiatum* (Zheng et al., 2017). This means that local differences in ECS volume or structural geometry could instead be the main determinants of putative variation in diffusional properties across these same layers, which has been reported, e.g., for the hippocampus (Arranz et al., 2014).

So far, the two-photon TR-FAIM approach has only been used by a single group, though it has a considerable potential for more discoveries, not least incorporated into a super-resolution modality, or in parallel with complementary techniques for addressing the ECS properties.

The technique comes with the advantages and disadvantages of conventional two-photon microscopy, with the additional trade-off that the required polarization-sensitive optical components will lower the photon-detection efficacy of the setup, so that effectively images will be less bright compared to conventional two-photon microscopy. When the involved fluorophore is applied extracellularly through injection or perfusion, this obstacle is quite readily circumventable as fluorophore concentration can simply be increased on the fly during the experiments.

## Super-Resolution Fluorescence Microscopy

### Super-Resolution Shadow Imaging

Super-resolution shadow imaging (SUSHI) is one of two main incarnations of super-resolution microscopy approaches developed specifically to address the geometric structure of the ECS in dense brain parenchyma (Tønnesen et al., 2018). Super-resolution microscopy encompasses several widely different fluorescent microscopy modalities that allow imaging beyond the diffraction barrier of light, which limits optical resolution in conventional microscopy approaches (Tønnesen and Nagerl, 2013). Among these, stimulated emission depletion (STED) microscopy stands out by being a point-scanning technique that optically enhances resolution, in two or three dimensions (3D), to directly produce super-resolved images (Klar et al., 2000). STED is based on either a respective confocal or two-photon microscope, with an additional and spectrally different laser beam co-aligned with the excitation beam to enhance resolution by restricting emission to a subdiffraction volume. In the most common confocal-based incarnation, the achievable optical resolution of 3D-STED inside live tissue is around 50 nm laterally and 150 nm axially, far superior to diffraction-limited microscopy techniques (Tønnesen et al., 2018).

**FIGURE 6:**
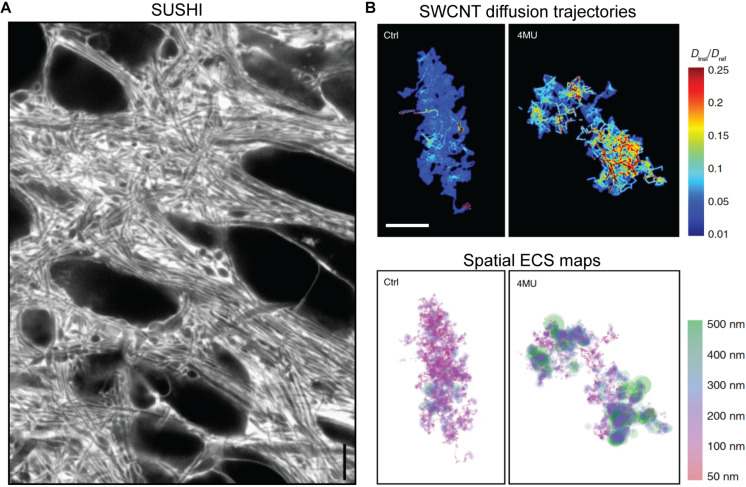
**(A)** Super-resolution shadow imaging (SUSHI) of the neuropil in cultured hippocampal slice. During perfusion of the slice with a fluorophore in solution, the interstitial fluid becomes brightly fluorescent, while cellular structures appear as dark shadows. As it is based on 3D-STED microscopy as the underlying imaging technique, it offers 50 nm planar resolution, which is sufficient to optically resolve the majority of ECS geometries in the frame. Scale bar is 4 μm. Modified from Figure 1E in Tønnesen et al. (2018), with permission. **(B)** Super-resolved maps of single-walled carbon nanotube diffusion trajectories (top) and corresponding ECS maps (bottom) in acute rat brain slices, with calibration bars indicating diffusional accesibility for SWCNTs in the ECS and derived ECS dimensions. The left panels are from control animals, while the right ones are from hyaluronan-depleted animals that were chronically administered 4-methylumbelliferone (4MU; a hyaluronan synthase inhibitior) prior to the experiment. Scale bar is 2 μm. From Soria et al. (2020), Nature Communications (scale bar inserted from cropped panel in the original figure), with permission (http://creativecommons.org/licenses/by/4.0/).

Just like two-photon shadow imaging described above (Kitamura et al., 2008), SUSHI is based on labeling of the interstitial fluid with a freely diffusible hydrophilic fluorophore that renders cellular structures visible as shadows. However, due to the several hundred times higher volume resolution (i.e., integrated planar and axial resolution of a microscope) of 3D-STED (Osseforth et al., 2014; Tønnesen et al., 2018), not only somata and dendrites become visible, but also structures of dense parenchyma, such as individual pre- and post-synapses, thereby generating unprecedented images of the entire neuropil (Figure 6A). Importantly, because the ISF compartment is literally identical to the ECS in terms of structural geometry, the ECS meshwork becomes directly visible and nearly completely optically resolved, allowing the ECS to be analyzed geometrically (Tønnesen et al., 2018).

As a point-scanning fluorescence microscopy modality, SUSHI is particularly well suited for time-lapse imaging inside live brain tissue, where it has been applied down to at least 50 μm depth in slices. The approach is readily combinable with concurrent imaging of conventionally labeled neural cells to reveal the ECS and neuropil in the context of individual neurons and glia cells (Tønnesen et al., 2018). It has provided the first live tissue quantitative geometric analyses of hippocampal ECS geometry and shown that ECS channel widths represent a continuous distribution spanning from microns down to 50 nm, with some unresolved structures likely even smaller. SUSHI has further revealed activity-dependent microscale ECS dynamics, though the involved cellular structures behind these remain to be identified. From the high-resolution images of the ISF, the ECS volume fraction can be calculated at any area of interest, down to the nanoscale volumes surrounding individual synapses.

Just like the related, but diffraction-limited, point-scanning modalities confocal and two-photon microscopy, the main limitation of the SUSHI approach is the imaging depth. The strict requirement for beam co-alignment and for shaping the STED beam wave front poses restrictions on the achievable imaging depth, though the achieved 50 μm depth of 3D-STED is already sufficient to be applicable in acute slices and *in vivo* in rodents. The effective imaging depth will undoubtedly improve in the near future by adopting emerging technologies from the two-photon and super-resolution microscopy field, including aberration correcting adaptive optics, implantable lenses, and more. In STED imaging, the requirement for an additional and proportionately high-power laser line introduces complexity, especially for multicolor imaging, and increases potential phototoxicity and bleaching effects, which may confound cellular imaging if not performed meticulously (Lenz and Tønnesen, 2019). However, the SUSHI configuration is practically insensitive to photobleaching and phototoxicity because bleached fluorophores simply diffuse away from the field of view and are not trapped inside the cells.

### Carbon Nanotube Localization Microscopy

A complementary super-resolution microscopy approach for investigating the ECS is based on imaging and tracking of near-infrared fluorescent nanometer-sized single-walled carbon nanotubes (SWCNTs) inoculated into the brain parenchyma *in vivo* and later observed in acute brain slices (Godin et al., 2017). After injection into the lateral ventricle, nanotubes passively spread through the brain parenchyma, penetrating the structure of the ECS according to their size. Their distribution at given time points after injection will therefore convey information about the diffusional properties of the ECS, as well as describing the involved ECS structure (Figure 6B). The diffusing fluorescent nanotubes can be tracked up to 100 μm tissue depth using a widefield microscopy approach at millisecond timescale, opening the possibility for *in vivo* studies of relatively large tissue areas. Analysis of time-lapse images with super-resolution single-particle-tracking algorithms yield real-time data on their dynamics in live tissue, thereby enabling not only nanoscale structure to be determined, but also local relative diffusivity determined from the instantaneous diffusion speed (Godin et al., 2017; Paviolo et al., 2020). This method has provided measurements of the ECS channel widths ranging from near 0 μm to around half a micron, describing a log-norm continuum with the most commonly observed width being just below 100 nm, in excellent agreement with the SUSHI study described above. It is noteworthy that fluorescent SWCNT tracking has suggested the ECS channel widths in organotypic hippocampal slices were slightly wider than in acute brain slices, though this needs to be more specifically addressed, as the organotypic slices were prepared from rat tissue, while the acute brain slices were prepared from mice, and the acute slice data were obtained from diverse regions that could potentially have different properties (Paviolo et al., 2020). This very basic question highlights the technical limitations that have hampered progress in the field, as well as the advantages of these latest methods.

The strength of the SWCNT imaging and tracking technique lies in providing both topological and diffusional information of the ECS simultaneously and enabling direct comparison between these two parameters. In a recent study that delivered the first nanoscopic characterization of pathological brain ECS, SWCNT tracking revealed poor correlation between local ECS width and nanoscale diffusion, suggesting that ECS diffusive inhomogeneities are not only driven by geometrical factors (Soria et al., 2020). In fact, SWCNT data from hyaluronan-depleted mice and mice with parkinsonian-like dopaminergic cell loss, which also induces hyaluronan degradation, showed that while matrix modification has a greater impact on molecular mobility, neurodegeneration affects both ECS channel width and diffusion (Soria et al., 2020). This interestingly suggests that hyaluronan, the main component of the extracellular matrix, is partially responsible for local variations in ECS diffusional properties.

Among the limitations of the technique is that ECS spaces smaller than the tubes will remain inaccessible and thus invisible and that tubes may conceivably get stuck and lose the ability to convey dynamic information, which further limits the potential for repeated experiments over time. A recent variation of the method, developed independently, makes use of fluorescent nanoparticles and multiple particle tracking algorithms to characterize diffusion in the ECS at single-particle resolution (McKenna et al., 2020). This approach confirmed increased nanoparticle diffusivity after matrix removal, although instead of hyaluronan as above, chondroitin-sulfate proteoglycan (CSPG) was modified by enzymatic digestion.

## Discussion

### Dog-Eat-Dog or Horses for Courses?

**FIGURE 7:**
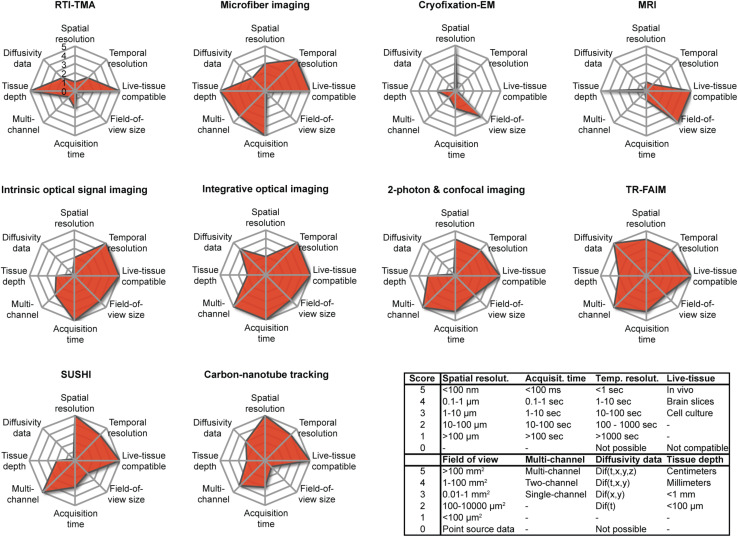
Overview of the parameters associated with the described modalities for investigating the brain ECS, as scored by us according to the inserted table using an arbitrary scale from 0 to 5. Note that the graphs are representative only of the current situation and that the parameters of a given technique may change and improve as a result of technical developments. The individual scores must be seen as estimates.

From their individual presentations above, and further by rating and comparing their main characteristics (Figure 7), it is clear that the various available techniques for addressing the brain ECS differ fundamentally in nature. They all have restriction in the sample they can be applied on and the type of data they provide, meaning that not all the above-described types of data can be collected from a given sample. In other words, no single technique is capable of extracting structural and biophysical data at high spatiotemporal resolution inside the intact live brain, which would be the ultimate experiment. Instead, each modality comes with particular limitations and particular strengths that limit the choice of techniques suitable for a given scientific question. In designing their experiments, investigators must carefully weigh the necessity for specific parameters, including spatial and temporal resolution, multicolor imaging, size of the field of view, and whether experiments must be live cell and repeated over time. Conversely, the availability of the techniques within an experimenter’s research institution or collaborative network may decide how a given scientific question can be addressed, if at all. Due to their widely differing nature, the techniques are not as much in competition with each other (dog-eat-dog scenario) as they are complementary, and each has its merit (horses-for-courses scenario).

If more than one method can be applied to a given sample, this can add synergy, as layers of variability can be peeled off during analyses to enable new insights. For example, knowing the relative diffusivity gradients across the hippocampal CA1 cell layer will allow stronger conclusions on how structure may shape diffusion across this layer and, accordingly, the determinants of tortuosity. Also, TR-FAIM should be readily doable using two-photon excitation STED microscopy in live brain slices or *in vivo* (Bethge et al., 2013; Takasaki et al., 2013), which would provide parallel nanoscale structural and diffusional data in a single acquisition step.

### New Discoveries on the Horizon

While a steady flow of publications, particularly from a few dedicated labs, have provided still deeper insights into the brain ECS structure and function, one frontier has remained inaccessible, namely the submicron ECS structure and its dynamics in live brain tissue. No method has existed that would provide credible structural images of the complex channels and reservoirs of the ECS in live brain tissue. The different nuclei and areas of the brain have hallmark organizations of neural cells into distinct layers and patterns, sometimes recognizable even to the naked eye, as for the hippocampus and striatum. This provides a convenient map for researchers, enabling them to quickly navigate the macrostructures of the brain, and even more so if individual cells are made visible through routine fluorescent labeling to bring them out in images. This mapping is still lacking for the ECS, and we have no information about its putative regional patterning across the areas of the brain. In other words, there is no ECS map to complement the existing cellular maps and, similarly, no regional ECS structure–function relationships attributed to specific brain areas.

We have referred frequently to the glymphatic system in this review, and several prominent questions pertaining to this are unanswered. Among the most prominent ones is the extent of ISF or CSF flow in the brain and the importance of this for metabolite clearance. Flow in the perivasculature does not equate flow in dense parenchyma, and indeed, the presence and extent of ISF flow in the ECS is currently being debated. Furthermore, it remains unknown how the overall circadian volume change of the glymphatic system is manifested across brain areas that differ greatly in terms of cell density and composition and across dense neuropil where structural changes may be very different from those of the immediate perivascular spaces. It similarly remains unknown which cell types mediate ECS dynamics at any scale, diurnal and others.

In this context, the appearance of the new super-resolution microscopy modalities, SUSHI and carbon nanotube tracking, can be considered a battering ram being moved into position, one that will inevitably open up access to new knowledge beyond this frontier. These techniques are further well suited for the all-important investigation of ECS structure and dynamics with respect to the organization of extracellular matrix molecules, as well as with respect to the accumulation of aggregating toxic protein species, such as prominent α-synuclein in Parkinson’s disease and β-amyloid plaques in Alzheimer’s disease, as reviewed in Irvine et al. (2008). Needless to say, these should be applied along the well-proven existing techniques, which will contribute in reciprocal validation, and putatively new unexpected biological insights.

The stage is set for near future breakthroughs in the brain ECS field, which will seed new research lines in fundamental brain physiology, as well as further translational studies in neurodegenerative disorders and beyond.

## Author Contributions

All authors listed have made a substantial, direct and intellectual contribution to the work, and approved it for publication.

## Conflict of Interest

The authors declare that the research was conducted in the absence of any commercial or financial relationships that could be construed as a potential conflict of interest.
